# Nocturnal nasal high-flow oxygen therapy in elderly patients with concomitant chronic obstructive pulmonary disease and obstructive sleep apnea

**DOI:** 10.1007/s11325-022-02702-2

**Published:** 2022-09-03

**Authors:** Lucia Spicuzza, Gianluca Sambataro, Matteo Schisano, Giuseppe Ielo, Salvatore Mancuso, Carlo Vancheri

**Affiliations:** 1 Department of Clinical and Experimental Medicine, University of Catania, Catania, Italy; 2 UO Pneumologia, Azienda Policlinico-San Marco, Via S. Sofia, 95123 Catania, Italy

**Keywords:** High-flow nasal cannula, Obstructive sleep apnea, COPD, Overlap syndrome, Nocturnal hypoxemia

## Abstract

**Purpose:**

The coexistence of obstructive sleep apnea (OSA) and chronic obstructive pulmonary disease (COPD) is known as “overlap syndrome” (OS). Patients with OS are usually older than patients with OSA alone, suffer from more profound oxygen desaturation during the obstructive events often accompanied by sustained nocturnal hypoventilation. Although oxygen-enriched positive airway pressure (PAP) is the treatment of choice in these patients, this therapy is often poorly tolerated particularly by the elderly. The aim of this study was to assess the usefulness of nocturnal oxygen therapy via nasal high flow (NHF-OT) as a possible alternative to PAP in patients with OS.

**Methods:**

Patients > 65 years old with OS and nocturnal respiratory failure (time spent below SaO_2_ 90% (T90) > 30%) had cardio-respiratory monitoring performed at baseline, during NHF-OT, or during conventional oxygen therapy (COT).

**Results:**

A total of 40 patients were enrolled in the study. NHF-OT significantly reduced the apnea–hypopnea index (AHI) in all patients compared to baseline and COT. The mean basal AHI was 25.4 ± 8.6. During COT and NHF-OT, the AHI was 19.4 ± 7 and 5.4 ± 4.6, respectively (*P* < 0.001) and 19 patients reached an AHI < 5 during NHF-OT. The mean nocturnal SaO_2_% was 86.2 ± 2.6 at baseline and at equivalent FiO_2_ it significantly increased to 91.8 ± 2.4 during COT and to 93.9 ± 2.5 during NHF-OT (*P* < 0.001). The T90% was 48.7 ± 20.1 at baseline, 16.8 ± 11.7 during COT, and 8.8 ± 8.0 during NHF-OT (*P* < 0.001).

**Conclusions:**

In elderly patients with OS, nocturnal treatment with NHF-OT significantly reduces obstructive episodes and improves oxygenation. As the treatment is generally well tolerated compared to PAP, NHF-OT may be a possible alternative therapy in this subgroup of patients.

## Introduction

Obstructive sleep apnea (OSA) and chronic obstructive pulmonary disease (COPD) represent major health problems being highly prevalent among the adult population [1]. The coexistence of the two diseases in a single patient has been known for long time and is commonly defined as “overlap syndrome” (OS) [2]. While OSA is generally characterized by intermittent nocturnal hypoxemia consequent to obstructive events, in patients with COPD chronic airway obstruction may cause stable hypoxemia during the day becoming more severe during sleep [2]. Patients with OS, compared to patients with OSA alone, suffer from more profound oxygen desaturation during the obstructive event and sustained nocturnal hypoventilation, particularly during rapid eye movement (REM) sleep [3]. Indeed, this condition deserves great attention being associated with a greater cardiovascular risk and burden of co-morbidities compared to either diseases in isolation, particularly in older adults [4]. Positive airway pressure (PAP), including continuous positive airway pressure (CPAP) and bilevel positive airway pressure (BPAP), is the treatment of choice for patients with OS, together with low-flow oxygen therapy if sustained nocturnal hypoxemia occurs [5, 6]. In these patients, nocturnal PAP improves symptoms and reduces cardiovascular events and mortality [6, 7]. However, it is well acquired that the long-term compliance to PAP, mainly explored in OSA, is consistently poor due to the discomfort of breathing through a mask with a positive pressure applied [5, 8]. In older adults with OSA, CPAP treatment has been associated with improved cardiovascular outcomes and survival rates [9]. Though, the largest study available on adherence to CPAP among older adults with OSA has shown that only 58% out of 3229 users were adherent to the treatment after 1 year [9]. Patients with OS are generally older than patients with simple OSA and with increasing age the number of hours of CPAP use decreases. [10]. In many cases, elderly patients with OS and nocturnal respiratory failure are treated only with low-flow oxygen, in order to avoid the discomfort of PAP to such patients who, due to the age and co-morbidities, have a limited life expectancy.

Oxygen therapy via a high-flow nasal cannula (NHF-OT) is an emerging technique first used to treat acute respiratory failure in adults and neonates [11, 12]. The device is designed to provide high flows through the nose with an optimal degree of heat and humidification and to maintain a stable fraction of inspired oxygen (FiO_2_). NHF has gained great attention among clinicians as it is better tolerated by the patients compared to PAP [11, 12]. When high flows are delivered through the nose, a small pressure is generated in the upper airways that seems sufficient to overcome pharyngeal critical pressure thus reducing the apneic events [13, 14]. In addition, NHF may improve the respiratory mechanic with a positive effect on gas exchange [11].

Therefore, the aim of this study was to assess the overall effect of nasal high flow on obstructive events and nocturnal respiratory parameters in elderly patients with concomitant OSA and COPD. In order to this, we compared standard nocturnal cardio-respiratory studies during NHF-OT or low-flow conventional oxygen therapy (COT) in patients with OS and nocturnal respiratory failure, who rejected treatment with PAP.

## Methods

### Study population

The study was conducted in the “Sleep Laboratory, Respiratory Unit, University of Catania (Italy),” from January 2019 to July 2021. We screened patients > 65 years old with OSA, concomitant COPD, and nocturnal respiratory failure. All patients presenting such characteristics were offered treatment with either CPAP or BIPAP and were invited to try the devices. We recruited only those patients who refused such treatment. Inclusion criteria were as follows: (1) age > 65 years; (2) diagnosis of OSA moderate to severe or mild disease with symptoms; (3) diagnosis of COPD of any degree of severity; (4) nocturnal respiratory failure defined as time spent below SaO_2_ 90% (T90) > 30%; (5) stability of COPD and absence of exacerbation in the last month; (6) acceptance and tolerance of NHF-OT.

Stable COPD was defined as the lack of acute symptoms or exacerbations in the last 4 weeks. Exclusion criteria were as follows: (1) presence of diurnal respiratory failure (PaO_2_ < 60 mm Hg at rest); (2) presence of any other sleep-associated disorder, including Cheyne-Stokes breathing; (3) inability to perform functional respiratory tests; (4) inability to sign consent. The study was conducted according to Good Clinical Practices and the Declaration of Helsinki, approved by local institutional review committees and written informed consent to collect data and to publish was obtained for all patients.

### Study protocol

The study was conducted during three consecutive nights. In the first study night, nocturnal cardiorespiratory polygraphy was performed at baseline (room air). During the second night, each patient was randomly assigned to nocturnal polygraphy during COT or NHF-OT (myAirvo™ 2 Humidified High Flow System, Fisher & Paykel Healthcare Ltd, Auckland, New Zealand). During the third night, patients previously monitored during COT were monitored during NHF-OT and vice versa. If problems occurred during the nocturnal recording, the monitoring was repeated the next night.

Before setting the NHF-OT parameters for the study night, each patients underwent a short trial to assess tolerance to this treatment. The flow set for each patient was the highest tolerated, starting from 30 up to 60 L/min. For each patient, the same FiO_2_ was used during the two study nights as one of the aims of this study was to compare conventional low flows vs high flows in improving nocturnal SaO_2_. The FiO_2_ to use overnight was set during the day using a Venturi mask, in order to increase awake SaO_2_ by 3–4%.

For each patient, we collected demographic and clinical data, performed lung function tests to confirm the diagnosis of COPD and arterial blood gas test. OSA-related symptoms were assessed, and the Epworth Sleepiness Scale was used to measure sleepiness.

#### Measurements

All patients underwent overnight in-lab cardiorespiratory polygraphy using a 6-channel Vital Night Plus (Vitalair, Italy). The records we analyzed automatically and manually according to standard criteria [15]. Obstructive apneas were defined as a drop in peak signal excursion > 90% of pre-event baseline with a duration > 10 s in the presence of abdominal and thoracic movements. Hypopneas were defined as a reduction in flow ≥ 30% with > 3% oxygen desaturation or arousal, in the presence of abdominal and thoracic movements [17]. We made diagnosis of OSA when the apnea–hypopnea index (AHI), which is the number or respiratory events per hour, was > 5. OSA was considered mild when AHI was ≤ 15, moderate when AHI was ≥ 30, and severe when AHI was > 30. We also evaluated the mean nocturnal SaO_2_ and T90, the level of desaturation during obstructive events (ΔSaO_2_), and the nadir SaO_2_. According to standard criteria, nocturnal respiratory failure was defined as a T90 above 30% of the recording time.

The diagnosis of COPD was obtained by the clinical history and confirmed by standard spirometry [17]. The severity of airflow limitation was classified according to the GOLD guidelines, with GOLD 1 defining mild obstruction and GOLD IV a very severe obstruction [18].

Arterial blood gas (ABG) analysis was performed at room air at baseline and in the morning after each study night.

### Statistical analysis

Data were analyzed using SPSS 20 software (StataCorp, College Station, TX, USA). Descriptive statistic was used for demographic data, which are presented as mean ± standard deviation (SD). The comparison between two groups was performed using an appropriate Student’s *t*-test. The comparison among three groups was performed by one-way analysis of variance (ANOVA). To compare two categorical variables, we used the chi-square test (χ^2^). *P* value < 0.05 was considered to indicate statistical significance.

## Results

### Study population

We recruited a total of 40 patients (22 males mean age 75.6 ± 7.0, mean BMI 29.3 ± 5.2). Demographic and functional data of the patients are shown in Table 1. All patients well tolerated the treatment with NHF-OT; there were no drop-outs and no side effects. During the nocturnal recording, all patients exhibited both obstructive apneas and hypopneas. Most of the patients (62%) had a moderate OSA with an AHI included between 15 and 30 (Table 1). In the group, 65% of the patients were included in GOLD stage 2 that is a moderate stage COPD, whereas only 3 patients had a very severe COPD (GOLD 4) (Table 1). In addition, reviewing patients’ CT scans we found that 14 patients (35%) had mild to moderate emphysema. Although according to inclusion criteria all patients had a PaO_2_ > 60 mmHg, 98% of them had a moderate hypoxemia with a mean PaO_2_ of 67.4 ± 6.2 mmHg. The mean PaCO_2_ was 43.7 ± 4.6 mmHg, and 11 patients (27%) were mildly hypercapnic.

**Table 1:** Demographic and respiratory functional data of the group

	Patients (*n* = 40)
Males (%)	22 (55)
Age, yrs	75.6 ± 7.0
Body mass index, kg/m^2^	29.3 ± 5.2
Current smokers (%)	14 (35)
PaO_2_, mmHg	67.4 ± 6.2
PaCO_2_, mmHg	43.7 ± 4.6
PH	7.40 ± 0.02
Awake SaO_2_, %	93.3 ± 2.5
FEV_1_, % predicted	58.4 ± 13.4
FVC, % predicted	75.8 ± 10.4
Severity of COPD	
GOLD 1, *n* (%)	0
GOLD 2, *n* (%)	27 (65.5)
GOLD 3, *n* (%)	10 (25)
GOLD 4, *n* (%)	3 (7.5)
Severity of OSA	
Mild, *n* (%)	4 (10)
Moderate, *n* (%)	25 (62.5)
Severe, *n* (%)	11 (27.5)
ESS	11.6 ± 4.6

Data are expressed as mean ± SD. *FEV*_*1*_, forced expiratory volume in 1 s; *FVC*, forced vital capacity; *GOLD*, Global Initiative for Chronic Obstructive Lung Disease; *ESS*, Epworth Sleepiness Scale

### Effect of nocturnal nasal high flow or conventional oxygen therapy

**Fig. 1:**
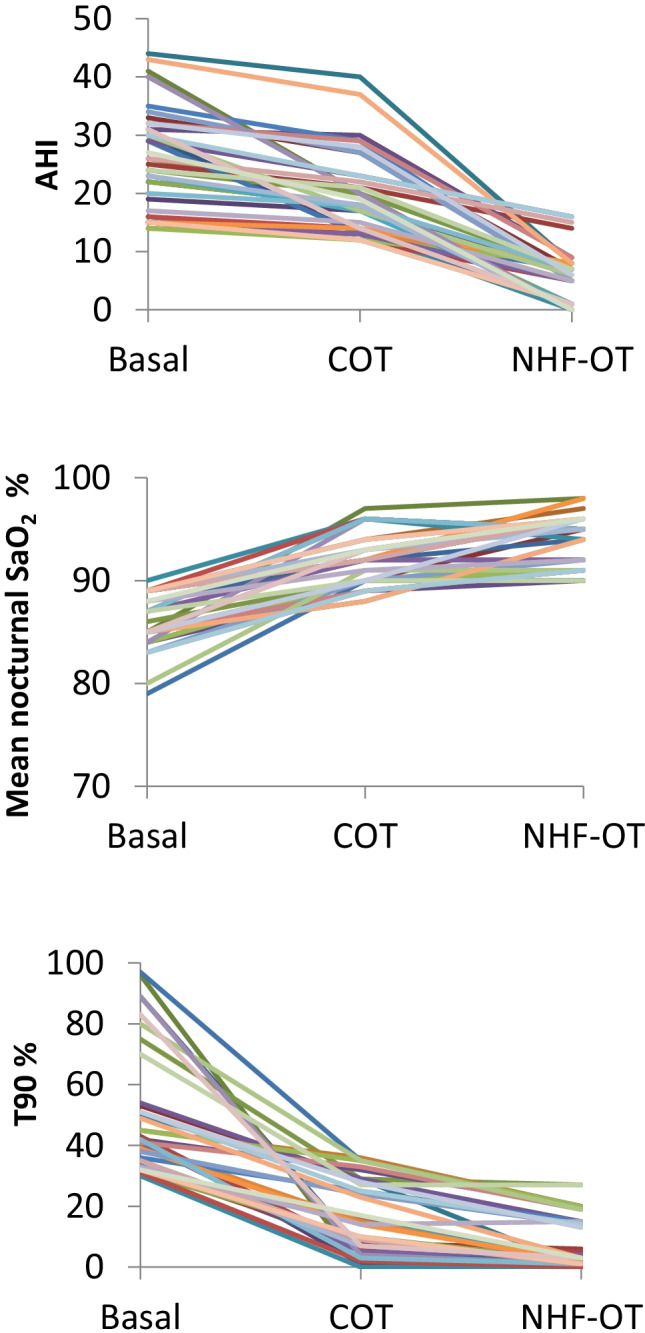
Changes in AHI, nocturnal mean SaO_2_, and T90 during COT and NHF-OT. AHI, apnea/hypopnea index; NHF-OT, nasal high-flow oxygen therapy; COT, conventional oxygen therapy

NHF-OT was administered with a mean flow rate of 46.3 ± 9 L/min (range 30–60). Although to a different extent, NHF-OT produced a significant reduction in AHI in all patients as compared to baseline and COT. In the whole group, the mean basal AHI was 25.4 ± 8.6 and was reduced to 19.4 ± 7 by COT and to 5.4 ± 4. 6 (*P* < 0.001) by NHF-OT (Table 2, Fig. 1). A response to NHF-OT was defined as an AHI ≤ 5, and 19 out of 40 patients were considered responders. However, it must be emphasized that in those patients with a suboptimal response the mean AHI during NHF-OT was 9.05 which is a remarkable improvement compared with a mean AHI of 26.4 recorded at baseline in this group (*P* < 0.001) (Table 3). An improvement in nocturnal SaO_2_ was obtained with COT and more markedly with NHF-OT. The mean nocturnal SaO_2_% was 86.2 ± 2.6 at baseline and at identical levels of FiO_2_ (mean value 27%) it significantly increased to 91.8 ± 2.4 during COT and to 93.9 ± 2.5 during NHF-OT (*P* < 0.001). Also the T90% changed from 48.7 ± 20.1 at baseline to 16.8 ± 11.7 during COT and 8.8 ± 8.0 during NHF-OT (*P* < 0.001) (Table 2). The ABG analysis performed at room air after each study night showed no significant differences in PaO_2_ or PaCO_2_ compared to baseline (Table 2). In order to understand the predictive factors for an efficacious response to NHF-OT, we analyzed differences among the groups of patients with AHI < 5 and those with AHI > 5 during NHF-OT. The two groups had similar age, BMI, severity of OSA distribution, similar hypopnea index, and similar prevalence of emphysema (Table 3). A small but significant difference was observed in diurnal and mean nocturnal SaO_2_ at baseline that were slightly higher in responders vs non-responders (Table 3). The mean flow rate set in NHF-OT in the group of responders was significantly higher as compared to the flow rate used for non-responders (50.6 L/min vs 42.7 L/min, *P* < 0.05) (Table 3).

**Table 2:** Changes in respiratory parameters during NHF-OT and COT

	Baseline	COT	NHF-OT	*P* value
AHI (events/h)	25.4 ± 8.6	19.4 ± 7.0	5.4 ± 4.6	< 0.001
CAI (events/h)	0.3 ± 0.01	0.4 ± 0.01	0.4 ± 0.01	0.9
HI (events/h)	15.2 ± 4.1	9.4 ± 3.0	1.4 ± 0.6	< 0.05
Mean nocturnal SaO_2_, %	86.2 ± 2.6	91.8 ± 2.4	93.9 ± 2.5	< 0.001
T90, %	48.7 ± 20.1	16.8 ± 11.7	8.8 ± 8.0	< 0.001
ΔSaO_2_, %	4.9 ± 0.8	3.7 ± 1.14	2.4 ± 1.0	< 0.001
Minimal nocturnal SaO_2_, %	75.8 ± 7.9	81.5 ± 7.4	84.1 ± 7.7	< 0.05
PaO_2_, mmHg	67.4 ± 6.2	67.8 ± 5.6	68.1 ± 4.8	0.9
PaCO_2_, mmHg	43.7 ± 4.6	44.2 ± 4.5	42.1 ± 5.8	0.3
PH	7.40 ± 0.02	7.39 ± 0.02	7.41 ± 0.02	0.8

Data are expressed as mean ± SD. *AHI*, apnea–hypopnea index; *CAI*, central-apnea index; *HI*, hypopnea index; *T90*, time below SaO_2_ 90%; *ΔSaO*_*2*_, change in SaO_2_ during the respiratory event. ABG was obtained in the morning at room air at baseline and after treatment with COT or NHF-OT

**Table 3:** Differences among responders (AHI < 5) and non-responders (AHI > 5) to NHF-OT treatment

	AHI < 5 (*n* = 19)	AHI > 5 (*n* = 21)	*P* value
Males, (%)	6 (31.5)	12 (57.1)	0.10
Age, yrs	75.1 ± 6.7	75.9 ± 7.5	0.81
Body mass index, kg/m^2^	29.8 ± 3.3	28.8 ± 5.7	0.20
FEV_1_, % predicted	56.3 ± 12	59.6 ± 5.7	0.30
Patients with emphysema (%)	6 (31.5)	8 (38.0)	0.51
Awake SaO_2_, %	94.5 ± 2.3	92.2 ± 2.1	< 0.05
AHI (events/h)	24.3 ± 5. 6	26.4 ± 8. 6	0.45
HI (events/h)	17.2 ± 2.1	14.2 ± 4.1	0.43
CAI (events/h)	0.3 ± 0.01	0.4 ± 0.01	0.87
Mean nocturnal SaO_2_, %	87.2 ± 2.1	85.2 ± 8.6	< 0.05
T90, %	45.6 ± 23	51.1 ± 16.5	0.35
NHF-OT flow, L/min	50.6 ± 8.8	42.7 ± 8.7	< 0.05

Data are expressed as mean ± SD. *AHI*, apnea/hypopnea index; *HI*, hypopnea index; *T90*, time spent below SaO_2_ 90%

## Discussion

This study showed that in older adults with overlap OSA-COPD with nocturnal respiratory failure, the application of nocturnal NHF-OT significantly reduced the number of obstructive events below a threshold that can be considered clinically significant or reduced the AHI to normal values. In addition, compared to conventional low-flow oxygen therapy, NHF-OT significantly improved nocturnal hypoxemia for the same set FiO_2_.

This is the first study on NHF on patients with overlap syndrome. Only few studies have previously explored the effect of nasal high-flow insufflation on upper airway obstruction [13, 14, 19, 20]. In 2007, an early physiological study showed that in 11 patients with mild to moderate OSA a transnasal airflow of 20 L/min improved the AHI, which changed from average 28 to 10, and reduced the number of arousals/hour from 18 to 8, thus improving sleep quality [13]. In order to explore the mechanisms responsible for this effect, the authors assessed the inspiratory airflow, end-expiratory supraglottic pressure, and respiratory effort during insufflation. It was found that after the insufflation was initiated there was an instantaneous increase in end-expiratory supraglottic pressure and inspiratory flow, thus suggesting that the relief of upper airway obstruction was most likely due to the small but consistent increase in pharyngeal pressure [13]. In a larger study including 56 patients with a wide spectrum of OSA severity, treatment with transnasal insufflation at 20 L/min during the night decreased the average respiratory disturbance index (RDI) from 22 to 17 [14]. A therapeutic response, defined as a reduction of RDI below 10 associated with a 50% reduction of the event rate from baseline, was observed in 27% of the patients [14]. In this study, there a greater response was observed in patients who predominantly had obstructive hypopneas or respiratory effort-related arousals and in patients who predominantly had REM-related events [14]. Although the increase in pharyngeal pressure during nasal insufflation has been pointed as the main mechanism preventing upper airway obstruction, the intraluminal pressure generated is low, so that other mechanisms, which remain largely unknown, could be involved. Although experimental evidence lacks, it has been suggested that another putative mechanism is the increase in lung volume produced by NHF that may contribute to increase upper airway patency [13, 21].

Other more recent studies evaluated the role of NHF as an alternative to CPAP using devices intended to generate flow rates up to 60 L/min. In one prospective study comparing NHF to CPAP, the flow rate of NHF was titrated during the night (from 20 to 60 L/min) similarly to the procedure used for CPAP [19]. Again, NHF significantly reduced the mean AHI from 34.9 to 14.8 at flow rates included between 40 and 60 L/min. NHF also significantly improved snoring indices and significantly increased the minimal SpO2. As expected, CPAP showed a more favorable effect on respiratory events and sleep efficiency [19]. Compared to previous studies, performed in younger patients with OSA, we found a more marked response in terms of AHI reduction since half of the patients had a reduction of AHI below 5 and the remaining patients a mean residual AHI of 9. This could be due, at least in part, by the higher flow rates used in our study compared to early reports (20 L/min). Moreover, this is confirmed by the fact that the analysis of subgroups of responders and non-responders showed that responders were those tolerating higher flow rates. Indeed the nasopharyngeal airway pressure generated by NHF increases with flow, changing from 1.5 to 3 cm H2O when the flow increases from 30 to 50 L/min [22]. Also the patients’ age could be a determinant of this good response to NHF. This is suggested by the study of Kostikas and co-workers who observed that the appropriate CPAP pressure required for elderly patients is, on average, 2.5 mmHg less than that used for younger people adjusted by OSA severity [23, 24].

Indeed COT is not a therapeutic option for patients with OSA, as it may prolong the duration of apneas although improving oxygenation [25, 26]. The use of COT in COPD with nocturnal desaturation, established many years ago by the NOTT group, has been more recently disputed, while no data are currently available for patients with overlap syndrome [25, 27]. In addition, there are no standard criteria to titrate nocturnal oxygen flow. In the clinical practice, repeated nocturnal monitoring with increasing FiO_2_ levels is usually performed to achieve results. Therefore, we chose empirically to use a level of FiO_2_ which was able to increase diurnal SaO_2_ by 3–4% (which is the physiological decrease in nocturnal SaO_2_). This level seemed appropriate as both COT and NHF-OT reversed nocturnal desaturation. However, we found that NHF-OT had a more favorable effect on nocturnal hypoxemia compared to COT, for the same set of FiO_2_.

A large amount of previous literature has focused on the benefit of high-flow oxygen therapy on the hypoxic respiratory failure, as compared to low-flow oxygen, particularly in the acute setting [11, 28, 29]. A number of mechanisms can explain this effect. One major mechanism is the delivery of the gas at flow rates exceeding the patient’s peak inspiratory flow rate. This allows a constant inspired FiO_2_ that is equivalent to the set FiO_2_ whereas the inspired concentration of oxygen at low flow is generally lower than the set FiO_2_ [28, 30]. In addition, the high flow produces a washout of the upper airways dead space, creating an oxygen reservoir within the upper airways [28, 31]. In patients with stable COPD, the benefits of NHF include the improvement of lung mucociliary clearance, the washout of upper airway dead space, the generation of a low level of positive airway pressure (PEEP effect), the decrease in inspiratory resistance, and, at the same time, an increase in the expiratory resistance [24]. Interestingly, in patients on long-term NHF-OT, together with a reduction in symptoms, it has been reported a subjective improvement in sleep quality [32, 33]. So far, data on the effect of NHF-OT on nocturnal hypoventilation in stable COPD are lacking and, as far as we know, this is the first study reporting a beneficial effect of this treatment on nocturnal hypoxemia.

In this study, we did not find any significant change in diurnal arterial blood gasses after treatment with NHF-OT, compared to baseline or COT. This may be due to the fact that one night is a short period to observe variations and, more likely, to the fact that patients were stable and normocapnic. In fact, it has been shown that in hypercapnic stable patients with COPD a short-term use of NHF-OT slightly reduced PaCO_2_ and improved oxygenation compared with COT [34]. In contrast, in normocapnic stable patients with COPD, treatment with NHF reduced the respiratory effort, without affecting blood gas levels [35]. These data were confirmed in a meta-analysis [36].

Data obtained from our study can help to identify elderly patients with OS as a subgroup responsive to nocturnal NHF-OT. One limitation of the study is that the sample was small, so that some predictive factors may have been missed, for example, the effect of gender. Another issue is that as physiological study, the effect of NHF was evaluated in a single night. One major issue is whether or not long-term application of this treatment, as an alternative to PAP, will reduce OSA-related symptoms, in particularly sleepiness, in order to improve the quality of life in these patients. Although we performed ESS at baseline, one night was a short period to observe change in sleepiness. Although these are elderly patients and life expectancy is limited compared to classical patients with OSA, the effect of NHF on OSA and COPD-related risks, mainly cardiovascular problems, is unknown and remains to be explored.

Indeed, the chronic use of NHF may have additional beneficial effects in patients with COPD. It is noteworthy that in obstructive pulmonary disease the inhalation of warm humidified air through NHF has been shown to improve mucus clearance [12]. Due to this and other physiological effects, the long-term NHF-OT is able to reduce exacerbations and hospitalization rate in patients with COPD [32]. So this may be another advantage of NHF-OT in OS, to be explored in long-term studies.

In conclusion, our data indicate that in elderly patients with overlap OSA-COPD and nocturnal hypoxemia who do not tolerate PAP, the nocturnal treatment with NHF oxygen therapy significantly reduces obstructive episodes and improves hypoxemia. As the treatment is generally well tolerated compared to PAP, NHF-OT may be an alternative therapy in this subgroup of patients. Further studies are needed to explore long-term benefits of this treatment.

## Data Availability

The datasets generated during and/or analyzed during the current study are available from the corresponding author on reasonable request.
